# Integrated Analysis of DNA Methylome and Transcriptome Reveals Regulatory Mechanism in the Longissimus Dorsi of Duroc Pigs

**DOI:** 10.3390/cells14110786

**Published:** 2025-05-27

**Authors:** Shiyin Li, Yarui Gao, Lixia Ma, Wei Chen, Zhao Ma, Zhanchi Ren, Yunzhou Wang, Yongqing Zeng

**Affiliations:** 1 Shandong Provincial Key Laboratory for Livestock Germplasm Innovation & Utilization, College of Animal Science and Technology, Shandong Agricultural University, Tai’an 271018, China; garyli1121@163.com (S.L.); 13721751403@163.com (Y.G.); wchen@sdau.edu.cn (W.C.); 15206902825@163.com (Z.M.); 17861218765@163.com (Z.R.); 2 Department of Veterinary Medicine, Shandong Vocational Animal Science and Veterinary College, Weifang 261061, China; 15053851361@163.com

**Keywords:** Duroc pig, skeletal muscle, WGBS, DNA methylation, *LPAR1*

## Abstract

DNA methylation plays a pivotal role in the epigenetic regulation of gene expression and holds promise for enhancing livestock meat production. In this study, we analyzed the DNA methylome and transcriptome of the longissimus dorsi muscle (LDM) in Duroc pigs with varying growth rates. Our results reveal that DNA methylation suppressed the expression of key muscle development markers (*MYOD*, *MYOG*, *MHC1*) and proliferation markers (*PI67*, *PCNA*), as well as the protein expression and phosphorylation of PI3K and AKT (*p* < 0.05). Dual-luciferase reporter assays and EMSA showed that SP1 overexpression enhanced the luciferase activity of the wild-type *LPAR1* promoter, an effect amplified by the demethylating agent 5-AZA (*p* < 0.05). The EMSA further demonstrates the relationship between SP1 and the *LPAR1* promoter region. Overexpression of SP1 upregulated *LPAR1* expression at both the mRNA and protein levels (*p* < 0.05). Knockdown of *LPAR1* reduced muscle marker gene expression and delayed myotube formation, while silencing *SP1* disrupted the expression of *LPAR1*, *MEF2C*, and *MHC1* (*p* < 0.05), and the demethylation induced by 5-AZA partially reversed these effects. These findings suggest that the DNA methylation/*SP1*/*LPAR1* axis is critical for skeletal muscle growth and development, underscoring the regulatory role of DNA methylation in muscle formation.

## 1. Introduction

Skeletal muscle tissue is composed of various cell types, including muscle fibers, adipocytes, endothelial cells, and connective tissue cells [1]. As the primary source of meat in livestock and poultry, the type, number, and diameter of muscle fibers directly influence meat yield and quality [2]. Consequently, the growth and development of muscle tissue in these animals are critical factors determining meat production.

DNA methylation is an epigenetic alteration in which a methyl group attaches to the fifth carbon of cytosine nucleotides [3], which plays a key role in the regulation of genes. This modification, particularly in the promoter areas and gene bodies, can result in persistent modifications of gene activity. It is vital for controlling gene functions during development and in a tissue-specific manner [4]. DNA methylation is also a key factor in cellular differentiation, tissue development, and the maintenance of genomic stability. In skeletal muscle, a highly differentiated tissue, DNA methylation is intricately involved in the regulation of gene expression networks and signaling pathways throughout its development [5,6]. Recent studies have highlighted the importance of DNA methylation in muscle development. For instance, DNA demethylation is known to activate the *MYF5*/*MYF6* super-enhancer [7], and the *PAX7* demethylation signature is critical for the acquisition and maintenance of muscle cell identity [8]. During myogenic differentiation, genes related to muscle development, such as *MYOD* and *MYF6*, are regulated by de novo DNA methylation [9]. However, the precise mechanisms by which DNA methylation regulates gene expression during skeletal muscle growth and development remain unclear.

Previous studies have primarily focused on methylation analyses across different pig breeds or during embryonic development in pigs [10,11]. In contrast, our study conducted an integrated methylome–transcriptome analysis of Duroc pigs (with full- and half-sibling relationships) during the late growth stage (110–130 kg) exhibiting distinct average daily gain (ADG). We systematically investigated the potential biological functions of DNA methylation during the post-developmental phase in swine. These findings provide novel insights into molecular processes regulating muscle growth and may hold significant application potential in terms of improving meat production in livestock breeding programs.

## 2. Materials and Methods

### 2.1. Animals

The Duroc pigs used in this study were sourced from a core breeding farm (with the informed consent of the animal owner). The pig herd included individuals ranging in body weight from 30 to 110 kg, with the top 30% of animals based on average daily gain (ADG). Performance measurements were continued until the pigs reached approximately 130 kg body weight. Based on ADG, eight pigs were selected and divided into two groups: the high ADG (H) group (774.89 g) and the low ADG (L) group (658.77 g). Each pair of high and low ADG pigs was either full siblings or half-siblings. All pigs were humanely slaughtered using electronic stunning followed by exsanguination at a local abattoir. Longissimus dorsi muscle (LDM) tissues were collected, immediately frozen in liquid nitrogen, and stored at −80 °C for future analysis.

### 2.2. RNA Extraction, Strand-Specific Library Construction, and Sequencing

Total RNA was extracted from LDM tissues with Trizol reagent kit (Invitrogen, Carlsbad, CA, USA) according to the instructions. RNA quality was assessed on an Agilent 2100 Bioanalyzer (Agilent Technologies, Palo Alto, CA, USA) and checked with RNase-free agarose gel electrophoresis. The enriched mRNAs were fragmented into short fragments with fragmentation buffer after ribosome RNA (rRNA) was removed. The first strand was transcribed with random primers. The second strand of cDNA was synthesized with DNA polymerase I, RNase H, dUTP, and buffer. Then, the cDNA was purified with QiaQuick polymerase chain reaction (PCR) extraction kit (Qiagen, Venlo, The Netherlands), end-repaired, poly(A) added, and ligated to Illumina sequencing adapters. Then, the second-strand cDNA was digested with uracil-N-glycosylase. The digested products were size selected with agarose gel electrophoresis, ampliffed, and sequenced with Illumina HiSeqTM 4000 (In this study, the paired-end sequencing method of Illumina sequencing was used, and each end was the 150 bp reads inserted into the target sequence, which were sequenced) by Gene Denovo Biotechnology Co., Ltd. (Guangzhou, China).

### 2.3. DNA Isolation, BS-Seq Library Construction, and Sequencing

High-quality genomic DNA was extracted from the LDM using DNeasy Blood and Tissue Kits (QIAGEN, Valencia, CA, USA). After genomic DNAs were extracted from the samples, DNA concentration and integrity were detected by NanoPhotometer^®^ spectrophotometer (IMPLEN, Westlake Village, CA, USA) and Agarose Gel Electrophoresis, respectively. Then, the DNA libraries for bisulfite sequencing were prepared. Briefly, genomic DNAs were fragmented into 100–300 bp by Sonication (Covaris, Woburn, MA, USA) and purified with MiniElute PCR Purification Kit (QIAGEN, Germantown, MD, USA). The fragmented DNAs were end-repaired and a single “A” nucleotide was added to the 3′ end of the blunt fragments. Then the genomic fragments were ligated to methylated sequencing adapters. Fragments with adapters were bisulfite converted using Methylation-Gold kit (ZYMO, Tustin, CA, USA), unmethylated cytosine is converted to uracil during sodium bisulfite treatment. Finally, the converted DNA fragments were PCR amplified and sequenced using Illumina HiSeqTM 2500 by Gene Denovo Biotechnology Co. Ltd. (Guangzhou, China).

### 2.4. Correlation of DNA Methylation and Gene Expression in Samples

To investigate the influence of DNA methylation on gene expression, genes were classified into four groups based on their expression levels, as determined by RPKM (reads per kilobase per million reads mapped): non-expressed group (RPKM≤1), low-expressed (1<RPKM≤10), middle-expressed (10<RPKM≤100), and high-expressed (RPKM>100).

Genes were divided into four groups according to their degrees of methylation: nonmethylated, low methylation, moderate methylation, and high methylation. This was performed in order to evaluate the effect of DNA methylation on gene expression. The remaining genes were evenly divided into three groups after the non-methylated group’s genes were eliminated. The statistical associations between DNA methylation and gene expression in the ±3 kb flanking regions and the gene body were assessed using Spearman’s correlation analysis. A positive correlation was indicated by rho>0, and a negative correlation by rho>0.

### 2.5. Correlation of DNA Methylation and Gene Expression Between H and L Groups

Differentially expressed genes (DEGs) (*p* < 0.05) were divided into four groups according to their expression patterns in order to examine the effect of DEGs on DNA methylation across groups: a special-down group (genes were specifically expressed in the L group), a special-up group (gene were specifically expressed in the H group), an other-down group (genes were reduced expression in the H group), and an other-up group (genes were increased expression in the H group).

Genes were categorized by genomic position, including the genebody area and the ±3 kb surrounding regions, in order to ascertain if the degree of DNA methylation in differentially methylated regions (DMRs) (*p* < 0.05) affects gene expression between groups.

### 2.6. Functional Enrichment Analysis of Differentially Methylated and Differentially Expressed Genes

To explore the potential functions of DNA methylation responsible for DEGs, gene ontology (GO) enrichment analysis and KEGG pathway enrichment analysis were conducted for DMEGs. The detailed information of differentially methylated and differentially expressed genes (DMEGs) can be found in Supplementary Table S5.

Firstly, all DMEGs were mapped to GO terms in the gene ontology database (http://www.geneontology.org/) accessed on 28 March 2022, and gene numbers were calculated for every term. Significantly enriched GO terms in genes compared with the genome background were defined by a hypergeometric test.

Genes usually interact with each other to play roles in certain biological functions. Pathway-based analysis helps to further understand genes biological functions. KEGG is the major public pathway related database (http://www.kegg.jp/kegg/) accessed on 28 March 2022.

### 2.7. RNA Interference and Overexpression of *LPAR1*

The oligonucleotides for RNA interference (RNAi) that target *LPAR1* and SP1 were synthesized and designed by Genepharma based in Shanghai, China. In order to create the *LPAR1* overexpression vector, the coding sequence of the mouse *LPAR1* gene was amplified utilizing forward and reverse primers that included EcoRI and XhoI restriction sites, respectively. Subsequently, the PCR products were ligated into the pcDNA3.1(+) vector provided by Sangon Biotech in China. The transfection of both the siRNA and the overexpression vectors was performed in C2C12 cells from Procell, China, to evaluate the impact of *LPAR1* on the differentiation of myoblasts. The sequences for the siRNA and primers can be found in Supplementary Table S1.

### 2.8. Cell Culture and 5-Aza Treatment

C2C12 cells were cultured in Dulbecco’s Modified Eagle’s Medium (DMEM), which is a widely used growth medium, sourced from HyClone in Logan, UT, USA. This medium was supplemented with 10% fetal bovine serum (FBS), an essential nutrient-rich component that supports cell growth and proliferation. The cells were maintained in a humidified incubator set at a temperature of 37 °C and containing 5% carbon dioxide (CO_2_), conditions vital for optimal cell culture. To initiate the process of myogenic differentiation, the standard culture medium was replaced with a specialized formulation of DMEM that included 2% heat-inactivated horse serum, referred to as differentiation medium (DM). This transition is crucial for promoting the development of muscle-specific characteristics in the C2C12 cells. The study aimed to evaluate the effects of 5-AzaC, a known inhibitor of DNA methylation, on both the proliferation and differentiation of myoblasts. C2C12 cells were first cultured in growth medium (GM) and subsequently treated with 5 μM of 5-AzaC, sourced from MCE in Monmouth Junction, NJ, USA, for a duration of three days. Following this treatment period, the cells were then transferred to differentiation medium for an additional four days. It is important to note that the culture medium was replenished every 24 h to maintain optimal conditions for cell growth and differentiation. Following the experimental treatment, total RNA and protein were extracted for further analysis, allowing for insights into the molecular changes that occurred during the treatment.

### 2.9. Quantitative Real-Time PCR

High-quality RNA was successfully extracted employing an RNA extraction kit provided by Tiangen, a well-known manufacturer based in China. Following the extraction, first-strand complementary DNA (cDNA) synthesis was carried out using the PrimeScript RT Reagent Kit from TaKaRa, a reputable company located in Japan. For the quantitative PCR (qPCR) analysis, specific primers were meticulously designed utilizing the Primer Premier 6.0 software, and these designed primers are detailed in Supplementary Table S1. The quantitative reverse transcription PCR (qRT-PCR) assays were conducted with precision in a reaction volume of 20 μL, using a Roche Light-Cycler^®^ 96 apparatus. TB Green was used as the fluorescent dye during the assays, adhering closely to the operational protocol outlined by the manufacturer, TaKaRa. To determine the relative RNA levels present in the samples, the comparative threshold cycle (Ct) method was employed, with β-actin serving as the internal normalization control to ensure the accuracy and reliability of the results obtained.

### 2.10. Western Blotting

After being extracted from different treatment groups, C2C12 cells were centrifuged for five minutes at a force of 12,000× *g*. As directed by the manufacturer, the cells were lysed using RIPA buffer (Beyotime, Shanghai, China) supplemented with 1 mM PMSF after centrifugation. The proteins were then moved onto a PVDF membrane after the resultant protein samples were resolved using SDS-PAGE on a 12% gel. The membrane was blocked for two hours at room temperature using a 5% skim milk (*w*/*v*) solution to avoid non-specific binding. To guarantee ideal binding, primary antibodies were then incubated for the whole night. The membranes were completely cleaned with PBST the next day, and primary antibodies were used to provide the best possible binding. To identify the target proteins, the membranes were completely cleaned with PBST the next day and then incubated with a secondary antibody for an hour at room temperature. The ChemiS-cope Analysis program (Clinx, Shanghai, China) was used to examine the protein bands. The investigation used primary antibodies against β-actin, LPAR1 (1:1000), AKT (1:1000), P-AKT (1:1000), MYOD (1:1000), MYOG (1:1000), and MEF2C (1:1000). Both primary and secondary antibodies were purchased from Bioss (Beijing, China).

### 2.11. Immunofluorescence Assay

After three washes with phosphate-buffered saline (PBS, pH 7.4), C2C12 cells were fixed in PBS containing 4% paraformaldehyde for 30 min. The cells were then permeabilized for 15 min in PBS supplemented with 0.2% Triton X-100. To block non-specific binding, cells or tissue slices were incubated with 5% (*v*/*v*) goat serum/PBS and 5% bovine serum albumin (BSA) for one hour at room temperature. Next, the cells were exposed to a primary antibody against MHC (1:100; R&D, Minneapolis, MN, USA) in 5% goat serum/PBS and incubated overnight at 4 °C for immunostaining. After washing with PBS, the cells were treated with Alexa Fluor 594-conjugated goat anti-mouse IgG1 (1:200; Zs-bio, Beijing, China) in 1% goat serum/PBS for two hours at room temperature. Nuclei were stained with 5 μg/mL DAPI (BioTime, Shanghai, China). Finally, the cells were washed three times with PBS and observed under a fluorescence microscope.

### 2.12. Luciferase Reporter Assay

HEK293 cells were plated in a 24-well plate and co-transfected with an SP1 overexpression plasmid (pcDNA3.1) and reporter vectors (pGL3-Basic) containing either the wild-type or mutant *LPAR1* promoter. The cells were then treated with 5-AzaC (MCE, Monmouth Junction, NJ, USA). After 48 h of transfection, luciferase activity was assessed using the Dual-Luciferase Reporter Assay System (Promega, Madison, WI, USA). The sequence of the LPAR1 promoter is provided in Supplymentary Table S1.

### 2.13. Electrophoretic Mobility Shift Assay

Biotin-labeled SP1 probes and unlabeled SP1 probes were synthesized (Beyotime, Shanghai, China), and the nuclear proteins were mixed with the labeled SP1 probes (with the biotin-unlabeled probes used as a specific competitor). For the supershift assay, an anti-SP1 antibody (Santa Cruz biotechnology, Dallas, TX, USA) was added to the mixture and incubated on ice for 30 min. DNA–protein complexes were separated by non-denaturing 5% PAGE in 0.5× Tris-borate-EDTA (TBE) buffer, followed by electroblotting onto a positively charged nylon membrane. After UV crosslinking, imaging was performed using the chemiluminescent EMSA kit (Beyotime, Shanghai, China) and analyzed with ChemiScope 6100 Analysis software (Clinx, Shanghai, China). The probe sequence is the SP1 consensus sequence, and its detailed information is provided in the Supplementary Table S1.

### 2.14. Statistical Analysis

The data were analyzed through one-way analysis of variance (ANOVA) by using statistical software SAS version 9.2. The results are expressed as means ± standard deviations (SD), with statistical significance defined as *, *p* <0.05, **, *p* <0.01, ***, *p* <0.001.

## 3. Results

### 3.1. Association Analysis Between Gene Expression and Methylation Rates Within the Sample

**Figure 1:**
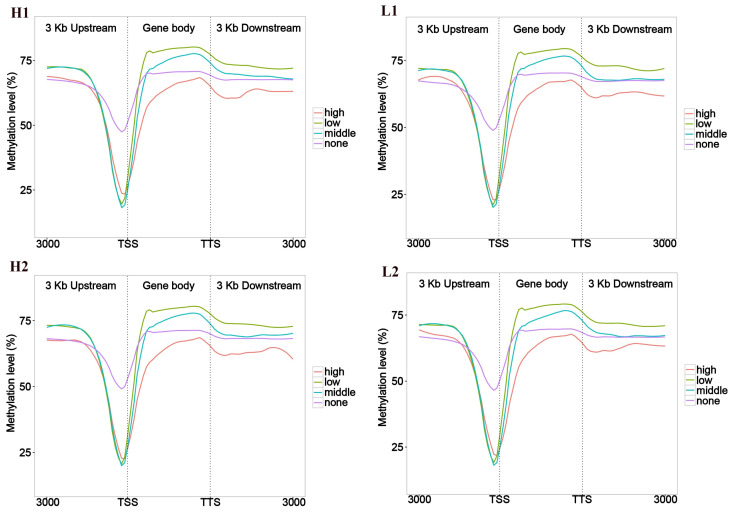

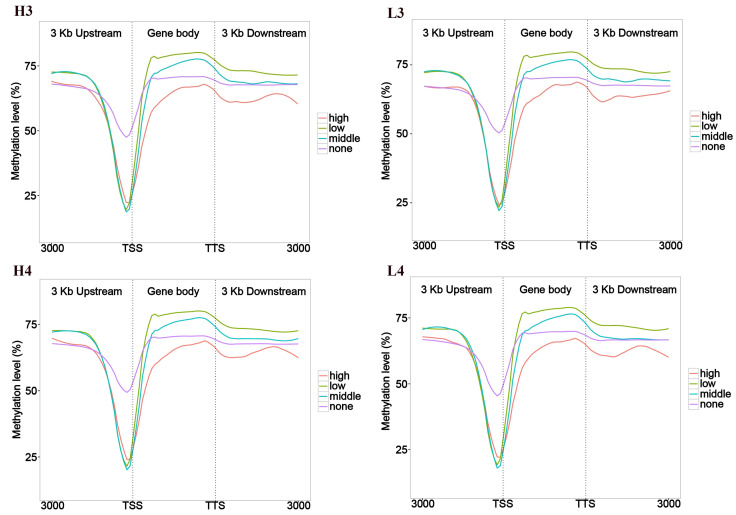
Map of different gene expression levels and DNA methylation levels. The x-axis represents the upstream and downstream positions of genes, with TSS and TTS indicating the transcription start site and transcription termination site, respectively. The y-axis represents the average methylation rate; different colors represent gene sets with varying expression levels. H1–H4: four Duroc pigs with high ADG; L1–L4: four Duroc pigs with low ADG.

**Figure 2:**
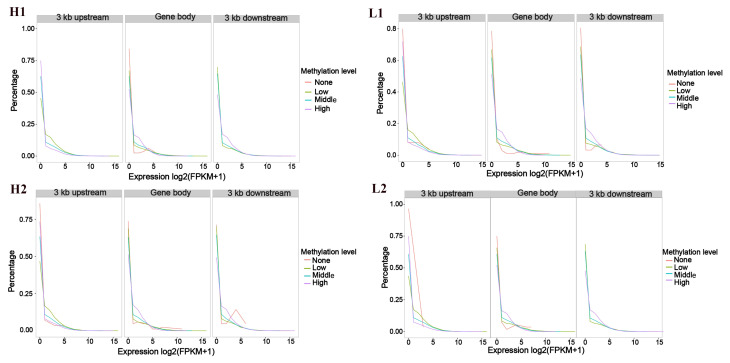

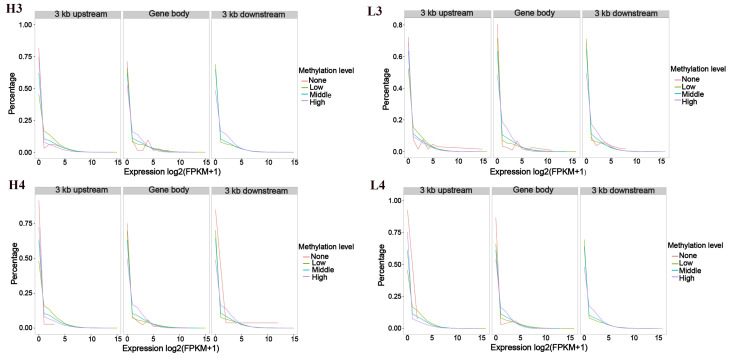
Frequency distribution of gene expression levels with varying methylation rates. The x-axis represents gene expression levels (log of FPKM values + 1), while the y-axis represents gene frequency. Different colors indicate varying methylation levels, with regions from left to right representing the upstream 3 kb region, gene region, and downstream 3 kb region. “None” refers to the non-methylated gene set. The methylated genes are divided into three average methylation level groups: “Low” for the low methylation gene set, “Middle” for the moderate methylation gene set, and “High” for the high methylation gene set. H1–H4: four Duroc pigs with high ADG; L1–L4: four Duroc pigs with low ADG.

Genes were classified into four groups based on their expression levels: non-expressed (FPKM ≤ 1), low expression (1<FPKM≤10), moderate expression (10<FPKM≤100), and high expression (FPKM >100). The average methylation rates were then calculated for each group in the gene body, as well as in the ±3 kb upstream and downstream regions. Our findings showed that gene clusters with high expression levels had lower methylation in the upstream, gene body, and downstream regions, while genes with low and moderate expression levels displayed higher methylation (Figure 1). In the upstream 3 kb region, most genes exhibited low methylation, while fewer genes exhibited high methylation. In contrast, a higher proportion of genes in the gene body and downstream regions exhibited high methylation, while a lower proportion showed low methylation. These results suggest that the upstream region tends to have a lower methylation, while the gene body and downstream regions are more likely to exhibit higher methylation (Figure 2).

### 3.2. Correlation Analysis of Gene Expression and Methylation Rates Between L vs. H Groups

**Figure 3:**
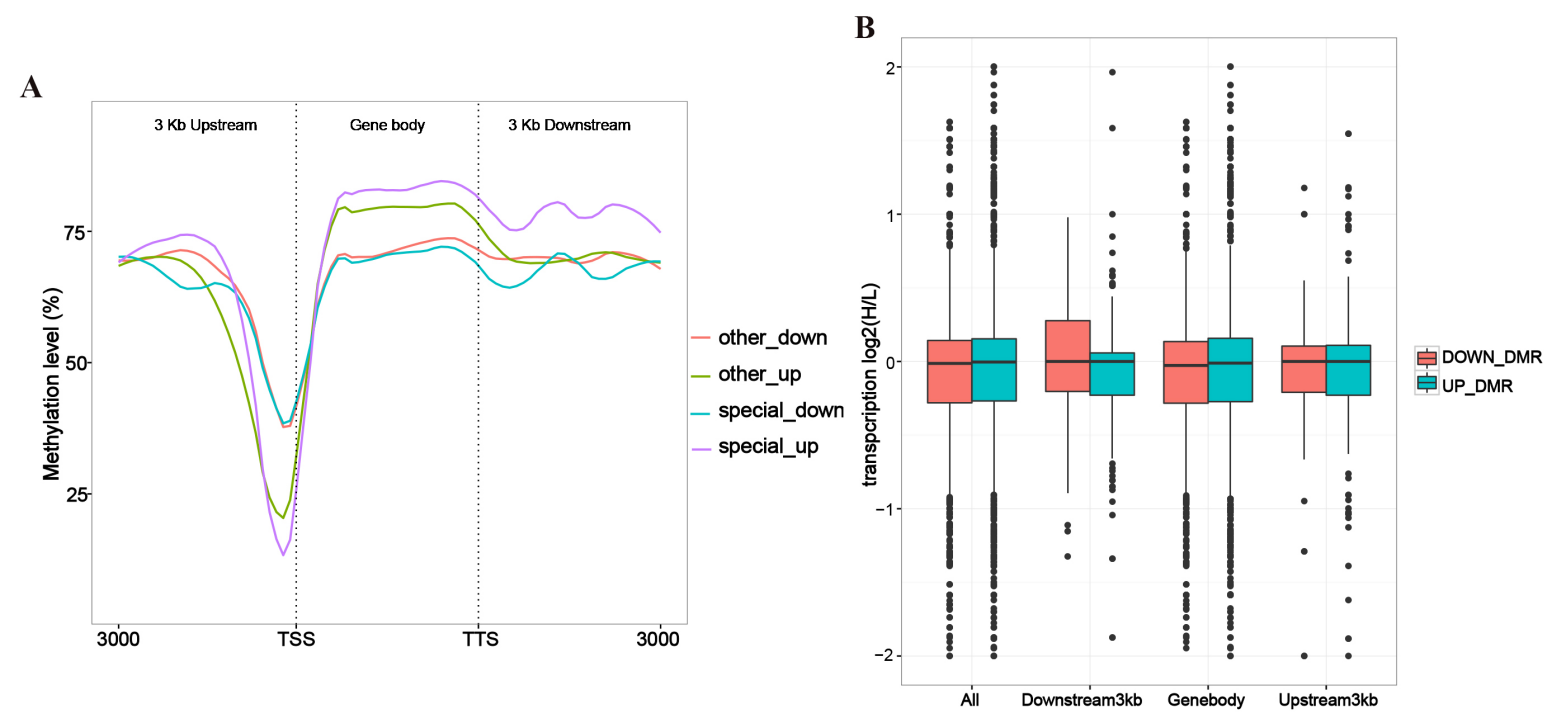
Analysis of differential gene expression and DNA methylation changes: (**A**) Analysis chart of differential gene expression levels and changes in methylation rates. Special up refers to genes that are specifically expressed in the H group but not expressed in the L group; special down refers to genes that are specifically expressed in the L group but not expressed in the H group; other up refers to non-specifically upregulated genes in the H group; and other down refers to non-specifically downregulated genes in the H group. The methylation level of DEGs in the H group (*n* = 4) vs. the L group (*n* = 4) are represented as the average value. (**B**) Analysis chart of DMR positions and changes in gene expression levels. The horizontal axis represents DMR positions, while the vertical axis represents the log of the fold change in gene expression. The gene expression levels in the H group (*n* = 4) vs. the L group (*n* = 4) are represented as the average value.

The relationship between differentially methylated regions (DMRs) and the positions of coding genes can influence the regulatory effects of DNA methylation. By classifying genes based on their positional relationship with DMRs, we can assess how variations in methylation levels across different regions impact gene expression and examine the patterns of expression changes in these genes. Our analysis revealed that in the gene body and the 3 kb downstream region, the overall methylation level was relatively high, ranging from 70% to 80%. Near the transcription start site (TSS), all methylation curves showed a significant decrease, with the lowest point around 20–30%. This pattern reflects the typical demethylation associated with gene activation. Furthermore, the lowest methylation levels observed near the TSS of the upregulated genes suggest that TSS demethylation facilitates the open conformation of the gene promoter region, promoting gene transcription (Figure 3A). Our study also found that the transcriptional level distributions of UP_DMR and DOWN_DMR in the overall DMR regions were similar, with both having a median close to 0, indicating no significant difference in transcription levels. However, the UP_DMR distribution was slightly higher. Additionally, a considerable number of outliers were observed in all regions, particularly those with log2 values close to −2, suggesting that some DMR regions may strongly suppress transcriptional activity (Figure 3B).

### 3.3. Analysis of Changes in Gene Expression Levels

**Figure 4:**
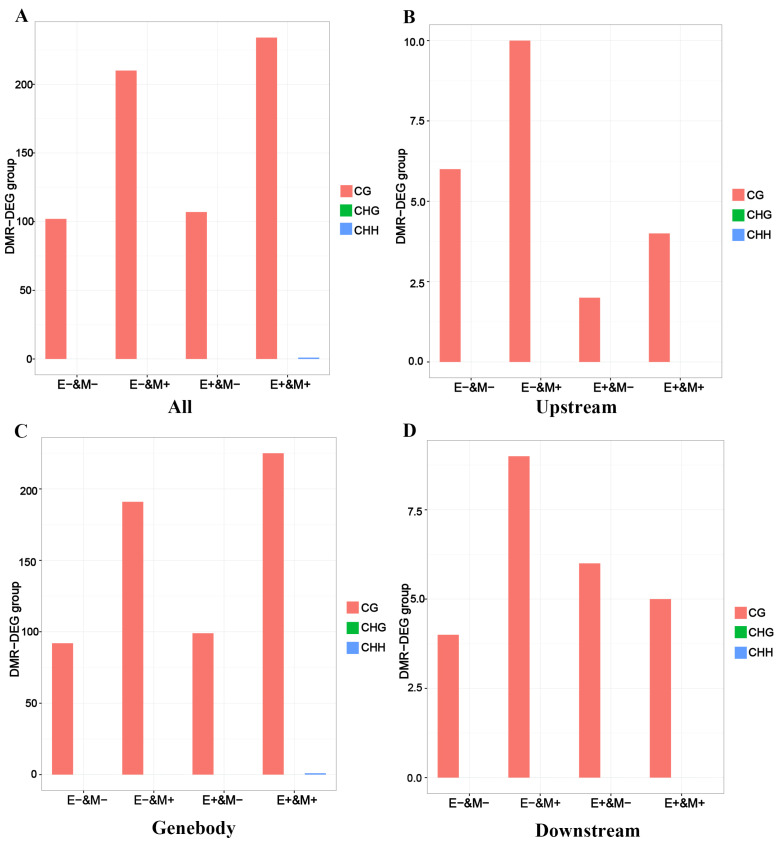
Bar chart of the changing trend of DMR-DEG groups (*n* = 4) expression levels: (**A**) The expression levels of DMR-DEG groups in DNA methylome and transcriptome. (**B**) The expression levels of DMR-DEG groups in the upstream region. (**C**) The expression levels of DMR-DEG groups in the genebody region. (**D**) The expression levels of DMR-DEG groups in the downstream region. E+/E− represents differentially upregulated/downregulated expressed genes; M+/M− represents differentially upregulated/downregulated methylated genes. CG, CHG, and CHH represent sequence contexts of DNA methylation sites.

In order to gain deeper insight into how DNA methylation interacts with gene expression during the later stages of growth and development in Duroc pigs, we conducted an extensive analysis of the genes associated with DMRs and DEGs (Supplementary Figure S1). Our analysis revealed that CG sites predominantly contributed to differences in gene methylation across all groups, with the highest numbers observed in the E− and M+ and E+ and M+ groups (Figure 4A). The number of DMRs and DEGs in the upstream regulatory regions of genes was relatively low. However, in the E− and M+ group, we observed some accumulation of CG methylation, suggesting that promoter region methylation may suppress transcriptional activity (Figure 4B). Conversely, the quantity of DEGs linked to CG methylation in the gene body region was notably greater than in other areas, particularly within the E+ and M+ group. This may be linked to the stability of methylation during transcription and its role in promoting transcription elongation efficiency (Figure 4C). Finally, methylation changes in the downstream region had a smaller impact on gene expression, likely because these regions are not directly involved in transcriptional regulation (Figure 4D).

### 3.4. GO Enrichment Analysis of DMEGs

**Figure 5:**
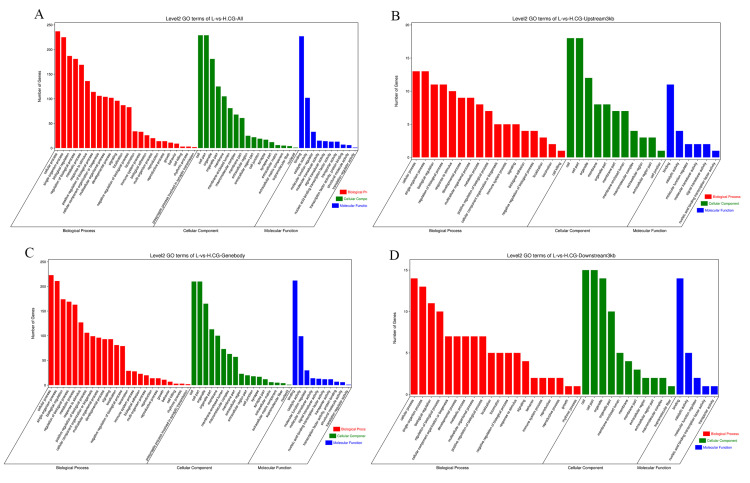
GO enrichment analysis of DMEGs in H vs. L groups: (**A**) GO enrichment analysis of DMEGs in genome-wide regions. (**B**) GO enrichment analysis of DMEGs in upstream regions. (**C**) GO enrichment analysis of DMEGs in genebody regions. (**D**) GO enrichment analysis of DMEGs in downstream regions.

Subsequently, to explore the potential functions of DMEGs, we conducted GO pathway enrichment analyses. Our results show that DMEGs were significantly enriched in several GO terms (Figure 5A, Supplementary Table S2 and Figure S2) related to the positive regulation of biological process (114), developmental process (102), binding (227), molecular function regulator (33), cell/cell part (229), organelle (181). Specifically, we found that the DMEGs were also significantly enriched in several GO terms associated with muscle development and function, including myofibril (10), actin cytoskeleton (17), sarcoplasm (5), muscle organ development (13), and muscle structure development (18). We also found that the number of enriched biological processes in the upstream region decreased (Figure 5B), which may indicate that methylation in the promoter region primarily affects a limited number of key biological processes. The enrichment level in the gene body region is similar to that at the whole-genome level (Figure 5C), suggesting that gene body methylation has broad regulatory functions. In contrast, the enrichment level in the downstream region is relatively small (Figure 5D).

### 3.5. KEGG Enrichment Analysis of DMEGs

**Figure 6:**
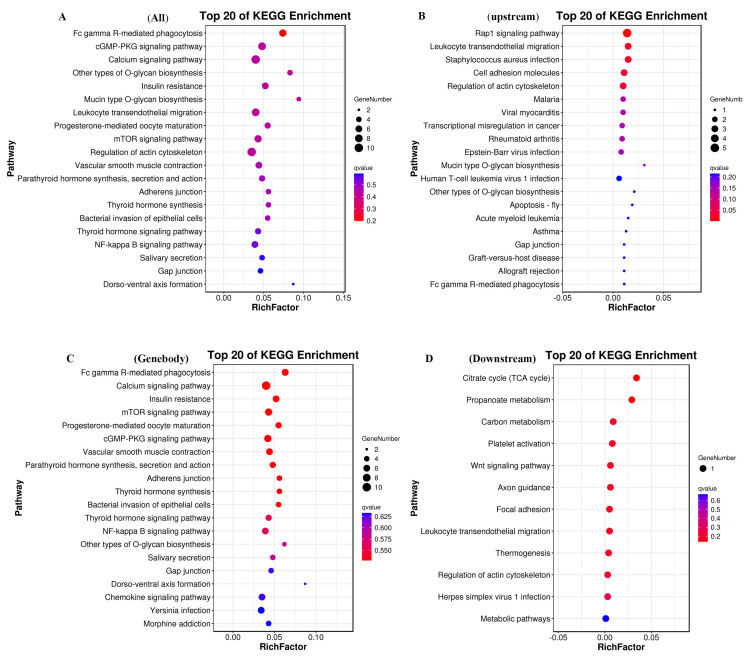
KEGG enrichment analysis of DMEGs in the H VS L groups: (**A**) KEGG enrichment analysis of DMEGs in genome-wide regions. (**B**) KEGG enrichment analysis of DMEGs in upstream regions. (**C**) KEGG enrichment analysis of DMEGs in genebody regions. (**D**) KEGG enrichment analysis of DMEGs in downstream regions.

Our results show that a number of DMEGs were enriched in several KEGG terms (Figure 6A, Supplementary Table S3). The five most highly represented pathways were Fc gamma R-mediated phagocytosis (7), the cGMP-PKG signaling pathway (8), the calcium signaling pathway (10), other types of O-glycan biosynthesis (4), and insulin resistance (6). We also identified several pathways associated with the growth and development of skeletal muscle. Specifically, in the upstream region, we observed a concentration of pathways involved in the regulation of Actin Cytoskeleton, NF-kappa B, and PI3K-Akt (Figure 6B). In the genebody region, we also found that pathways related to calcium signaling, insulin resistance, and mTOR signaling were enriched (Figure 6C). Additionally, in the downstream region, our results show the Wnt signaling pathway was enrichment (Figure 6D). These findings indicate that DNA methylation might contribute significantly to skeletal muscle growth and development.

### 3.6. DNA Methylation Influences the Development of Skeletal Muscle Through Its Impact on Gene Expression

**Figure 7:**
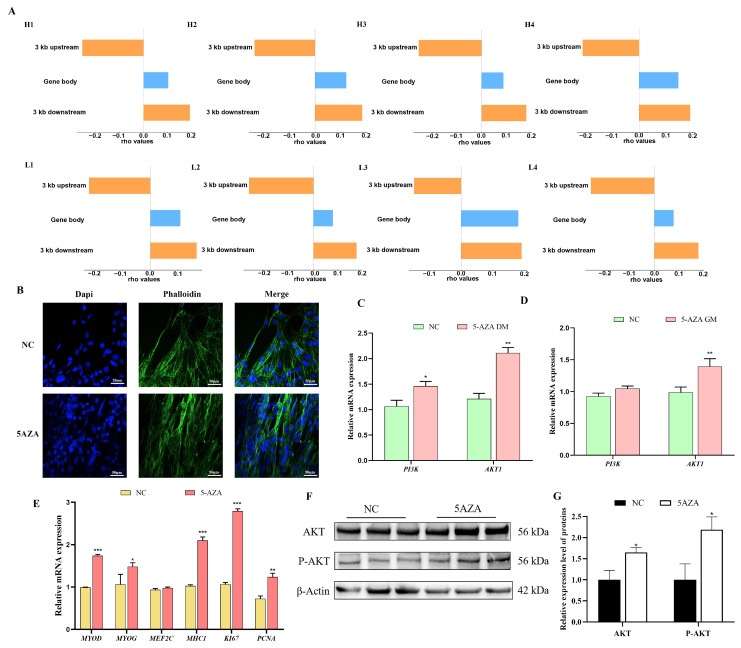
The role of DNA methylation on gene expression: (**A**) Correlation analysis chart of gene expression levels and DNA methylation levels. Rho refers to Spearman’s correlation coefficient. Rho > 0 indicates a positive correlation, while rho < 0 indicates a negative correlation. (**B**) The formation of the actin cytoskeleton in C2C12 cells treated with 5-AZA. The magnification of the picture is 20×, that is, the magnification of the objective lens is 20 times. (**C**,**D**) The mRNA expression level of PI3K AKT in C2C12 cells treated with 5-AZA. (**E**) Expression levels of proliferation and differentiation marker genes treated with C2C12 cells. (**F**) Western blot showing the key node gene (*AKT*) of the PI3K-AKT signaling pathway in C2C12 cells treated with 5-AZA. (**G**) Relative expression level of AKT/P-AKT proteins. The data are shown as the mean ± SD (*n* = 3), *, *p* <0.05, **, *p* <0.01, ***, *p* <0.001.

In order to explore the impact of DNA methylation on muscle development, we conducted a Spearman correlation analysis to assess the relationship between DNA methylation levels and gene expression in the 3 kb regions upstream and downstream of the coding genes. This approach allowed us to visualize the association between DNA methylation and gene expression across various genomic regions. The results indicate that the correlation between DNA methylation and gene expression differs depending on the region. More specifically, in the upstream 3 kb region, DNA methylation showed an inverse correlation with gene expression, while in the gene body and downstream regions, a positive correlation was observed (Figure 7A). We further explored the impact of DNA methylation on actin cytoskeleton formation by first determining the time required for its formation and confirming that treatment with the methylation inhibitor 5-AZA suppressed DNA methyltransferase expression (Supplementary Figures S3 and S4). To further assess the effect of DNA methylation on muscle development, we conducted FITC-conjugated cytochalasin staining and 5-AZA-induced demethylation treatment on C2C12 cells. The results indicate a significant increase in F-actin content in C2C12 cells following 5-AZA treatment (Figure 7B, Supplementary Figure S5). Additionally, the qPCR analysis revealed that 5-AZA-induced demethylation significantly increased the mRNA expression levels of *PI3K*, *AKT*, and myogenic markers (*MYOD*, *MYOG*, *MHC1*) during the differentiation period (DM). However, during the growth and proliferation period (GM), only the mRNA expression levels of AKT and proliferation markers (*KI67*, *PCNA*) were significantly elevated (Figure 7C–E). Western blot analysis further confirmed that 5-AZA-induced demethylation significantly increased both the phosphorylation and expression of AKT (Figure 7F,G). These findings suggest that DNA methylation plays a crucial role in inhibiting muscle formation, likely through the suppression of the PI3K-AKT signaling pathway during myogenic differentiation and proliferation.

### 3.7. DNA Methylation Regulates *LPAR1* by Modulating SP1 Binding in Skeletal Muscle

**Figure 8:**
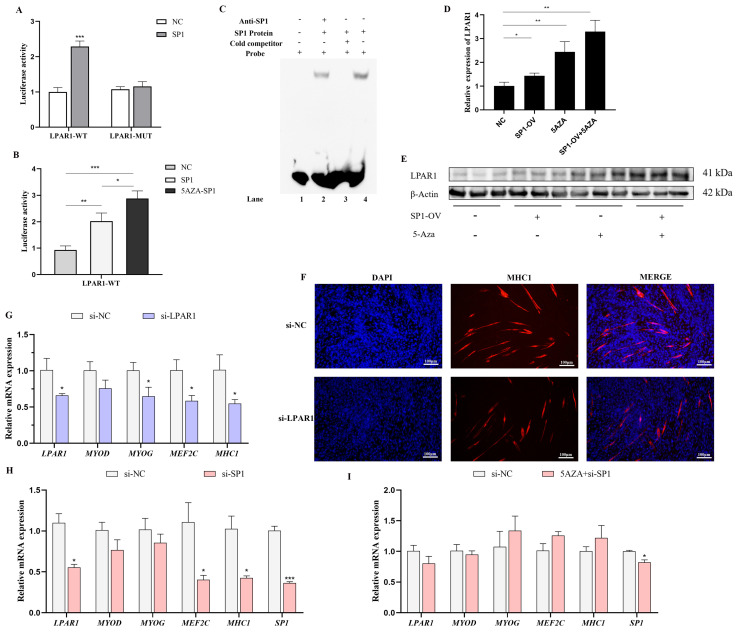
DNA methylation regulates gene expression by regulating the accessibility of TF binding: (**A**) Relative dual luciferase report for adding SP1 or NC to wild-type or mutant *LPAR1* (transfected cells are HEK293). (**B**) Report of relative double luciferase after the 5-AZA treatment of HEK293 cells. (**C**) The specific binding of *LPAR1* to SP1 was identified by Electrophoretic Mobility Shift Assay (EMSA). (**D**) The relative mRNA expression level of *LPAR1* after the overexpression of SP1 and 5-AZA-induced demethylation in C2C12 cells. (**E**) The Western blot showing the protein expression levels of *LPAR1* in C2C12 myoblasts after 5-aza-induced demethylation and SP1 overexpression. (**F**) Immunofluorescence detection of C2C12 myoblast differentiation after *LPAR1* gene knockdown. The magnification of the picture is 10×, that is, the magnification of the objective lens is 10 times. (**G**) Relative mRNA expression level of myogenic marker genes after knockdown of the *LPAR1* gene. (**H**,**I**) Relative mRNA expression levels of myogenic marker genes after SP1 gene knockdown and 5AZA-induced demethylation in C2C12 cells. The data are shown as the mean ± SD (*n* = 3), *, *p* <0.05, **, *p* <0.01, ***, *p* <0.001.

DNA methylation influences gene expression by regulating the accessibility of upstream transcription factors [12,13]. Based on our previous findings [14], we predicted the sequence of the differentially methylated region (DMR) upstream of *LPAR1* and identified an SP1 binding site (Supplementary Table S4). To investigate the interaction between SP1 and *LPAR1*, we performed dual-luciferase reporter assays and EMSA. Our results demonstrate that transfection with an SP1 overexpression vector significantly increased the luciferase activity of the wild-type *LPAR1*. Furthermore, treatment with 5-AZA further enhanced the effect of SP1 on *LPAR1*. In contrast, no effect was observed on the luciferase activity of the mutant *LPAR1* (Figure 8A,B). The EMSA further demonstrates the relationship between SP1 and the *LPAR1* promoter region (Figure 8C). Incubation of nuclear extracts with biotin-labeled SP1 probes resulted in the formation of DNA–protein complexes (Lane 4). When cold probes were included in the reaction, the number of DNA–protein complexes decreased (Lane 3). Moreover, the addition of an SP1-specific antibody reduced the number of DNA–protein complexes (Lane 2). These findings suggest that SP1 enhances the transcriptional activity of *LPAR1* by binding to its promoter region. Moreover, the hypomethylated state likely promotes the accessibility or open conformation of the *LPAR1* promoter, further strengthening this effect. The overexpression of SP1 resulted in an upregulation of both the mRNA and protein levels of *LPAR1*, and this effect was amplified by demethylation induced by 5-AZA (Figure 8D,E). Additionally, overexpressing *LPAR1* significantly promoted myogenic differentiation (Supplementary Figure S6). Conversely, interference with *LPAR1* expression led to the downregulation of myogenic marker genes and delayed myotube formation in myogenic cells (Figure 8F,G). Similarly, interference with SP1 expression significantly affected the mRNA expression levels of *LPAR1*, *MEF2C*, and *MHC1*. Notably, 5-AZA-induced demethylation alleviated this effect (Figure 8H,I). Finally, 5-AZA-induced demethylation and overexpression of SP1 significantly promoted the myogenic differentiation process. (Supplementary Figure S7).

## 4. Discussion

In this study, we conducted an integrated analysis of the methylome [14] and transcriptome [15] of the LDM in Duroc pigs with varying ADG, specifically focusing on full- and half-siblings during the 110–130 kg growth stage. This study plays a vital role in elucidating the epigenetic functions of DNA methylation during the advanced stages of livestock growth and meat production. The results offer an in-depth understanding of how DNA methylation potentially contributes to the growth and development of skeletal muscle.

Our analysis of the methylome and transcriptome highlights the role of DNA methylation across different genomic regions and its relationship with gene expression. Specifically, we observed that genes in the upstream regions tend to exhibit higher methylation levels when expressed at lower levels, while methylation in the gene body and downstream regions correlates positively with gene expression. These findings are consistent with previous studies [16,17,18]. To further explore the impact of differentially methylated regions (DMRs) on gene transcription, we performed an association analysis between gene expression and methylation levels. While overall changes were modest, we identified specific regions that may either activate or suppress gene expression. Previous research has demonstrated that DNA methylation in gene promoters can regulate chromatin structure and transcription factor binding, thereby silencing gene expression [19,20]. Although numerous studies have confirmed the impact of gene body DNA methylation on gene expression, its precise function remains largely unclear [21,22,23,24]. DNA methylation within the gene body is associated with gene silencing via chromatin condensation and its interactions with the transcribed regions [25,26,27]; other studies suggest that gene body methylation can also promote gene expression by regulating transcription and chromatin accessibility [28,29,30]. These results indicate that the role of DNA methylation in gene regulation depends on both the region and the methylation state, underscoring the need for further investigation into how methylation patterns influence transcriptional activity.

During muscle development, DNA methylation regulates genes involved in muscle cell proliferation, differentiation, and hypertrophy [27,31,32]. Several studies have shown that DNA methylation patterns in muscle-specific genes correlate with the muscle growth rate and meat quality in animals [33,34]. For example, genes such as *MyoD* and *IGF2*, which are crucial for muscle differentiation and growth, are regulated by DNA methylation. Changes in their methylation status can affect the muscle fiber composition and growth rate [35,36]). Our study also found that DNA methylation influences the expression of *MYOD*, *MYOG*, and *MHC1* during myogenic differentiation, thus affecting muscle cell growth and development. Additionally, our results emphasize the role of methylation in the PI3K-AKT signaling pathway, a key pathway during muscle cell proliferation and differentiation. Previous research has shown that the activation of the PI3K/AKT/mTOR pathway promotes myogenic differentiation and muscle formation [37]. Furthermore, the activation of the PI3K/Akt signaling pathway increases the expression and phosphorylation of PI3K and Akt [38,39,40]. In our study, demethylation significantly promoted the expression of PI3K and AKT, as well as their phosphorylation, suggesting that methylation inhibits activation of the PI3K / AKT signaling pathway, thus influencing the formation of skeletal muscles.

Previous studies have demonstrated that DNA methylation can inhibit regulatory elements, such as enhancers and promoters, to control gene transcription [41,42,43,44]. Our study indicates that DNA methylation modulates gene expression in skeletal muscle development through the regulation of SP1 binding to *LPAR1*. Importantly, prior research from our lab identified *LPAR1* as a critical gene linked to muscle growth, revealing a negative correlation between its promoter methylation and gene expression [14]. *LPAR1* plays a critical role in myogenesis and may participate in signal transduction during muscle differentiation [45,46]. In bovine satellite cells, *LPAR1* expression is upregulated during early differentiation, and its ligand can effectively induce myogenic differentiation [47]. Additionally, a GWAS analysis revealed a link between *LPAR1* and meat quality in Laiwu pigs [48]. This study demonstrates that DNA methylation negatively regulates the expression of *LPAR1* by modulating SP1 binding, highlighting the regulatory mechanism of DNA methylation during skeletal muscle development. Consequently, *LPAR1* stands out as a promising target gene for enhancing pork production traits and muscle development in pigs.

Recent research has demonstrated that not only does DNA methylation control the binding of transcription factors to various regulatory elements, including enhancers, it is also affected by histone modifications. This underscores the intricate and dynamic relationship between epigenetic changes and gene expression regulation [49,50,51,52]. As epigenetic editing technologies continue to evolve, research on dynamic changes in DNA methylation and epigenetic regulation during skeletal muscle growth may require emerging epigenetic tools for targeted studies [53,54].

## 5. Conclusions

In conclusion, this study underscored the critical role of DNA methylation in the regulation of skeletal muscle development. By integrating the methylome and transcriptome, we provide a comprehensive resource for understanding the specific contributions of DNA methylation to muscle development. This resource also serves as a valuable reference for research on animal genetic breeding, livestock meat production, and muscle growth. With the ongoing advancement of gene editing technologies, single-cell sequencing, and high-throughput epigenetic analysis, the future integration of these data is expected to deepen our understanding of how DNA methylation influences meat production performance. This will enable the optimization of the expression of genes related to meat quality, muscle development, and fat deposition, ultimately improving the production efficiency of livestock and poultry.

## Data Availability

RNA-Seq: https://www.ncbi.nlm.nih.gov/bioproject/?\term=PRJNA812354 (accessed on 6 June 2023). WGBS Data: https://dataview.ncbi.nlm.nih.gov/object/\PRJNA1054366?reviewer=onq3123u4ko2mkf270m5efipsq (accessed on 6 June 2023).

## Supplementary Materials

The following supporting information can be downloaded at https://www.mdpi.com/article/10.3390/cells14110786/s1, Figure S1: Clustering heatmap of DMEGs expression levels; Figure S2: GO enrichment analysis of DMEGs; Figure S3: The formation of the cytoskeleton; Figure S4: Changes in the expression levels of methyltransferases after 5-AZA treatment of cells; Figure S5: Quantification of Figure 7B; Figure S6: Overexpression of LPAR1 promotes differentiation of C2C12 myoblasts; Figure S7: Overexpression of SP1 and 5-AZA treatment promote myogenic differentiation in C2C12 cells; Table S1: Primer information used in the study; Table S2: GO enrichment analysis of DMGs and DEGs in H VS L groups; Table S3: KEGG enrichment analysis of DMGs and DEGs in H VS L groups; Table S4: Transcription Factor SP1 Binding Site Prediction; Table S5: The quantity of DMEGs.
