# Supplementary material for: Integrated Analysis of DNA Methylome and Transcriptome Reveals Regulatory Mechanism in the Longissimus Dorsi of Duroc Pigs
Source: Cells. 2025 May 27;14(11):786. doi: 10.3390/cells14110786 (PMC12153604; doi:10.3390/cells14110786)
Supplement: Supplementary file 1 [file cells-14-00786-s001.zip › cells-3603463-supplementary.pdf]

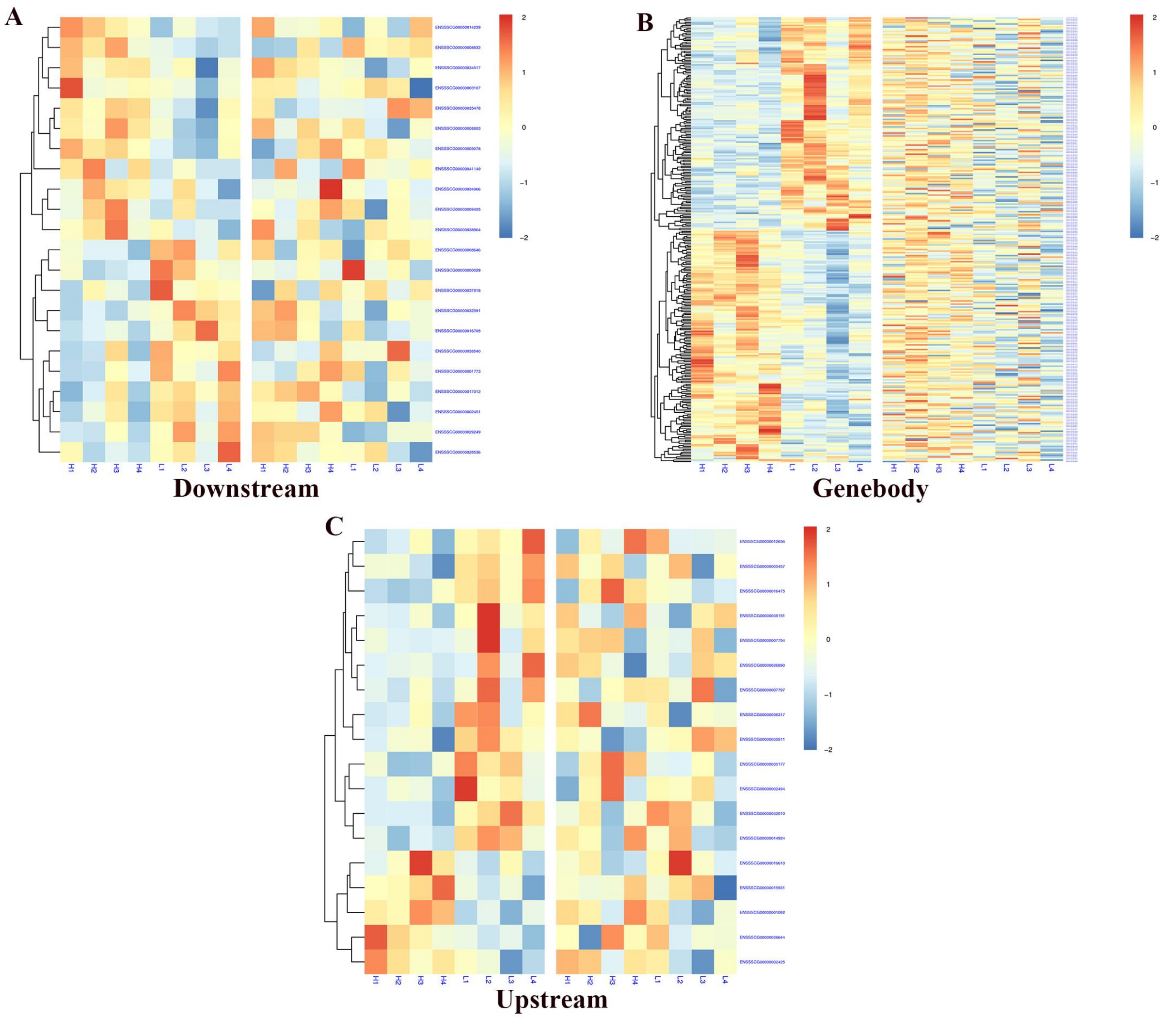

Supplementary Figure S1. Clustering heatmap of DMEGs expression levels. A Clustering heatmap of DMEGs expression levels in the downstream region. B Clustering heatmap of DMEGs expression levels in the gene body region. C Clustering heatmap of DMEGs expression levels in the upstream region. The left panel shows a heatmap where each column represents a sample, and each row represents a gene. The expression levels of the genes in different samples are represented by different colors, with red indicating higher expression and blue indicating lower expression. The right panel shows a heatmap where each column represents a sample, and each row represents a gene. The methylation levels of DMR-related genes in different samples are represented by different colors, with red indicating higher methylation and blue indicating lower methylation

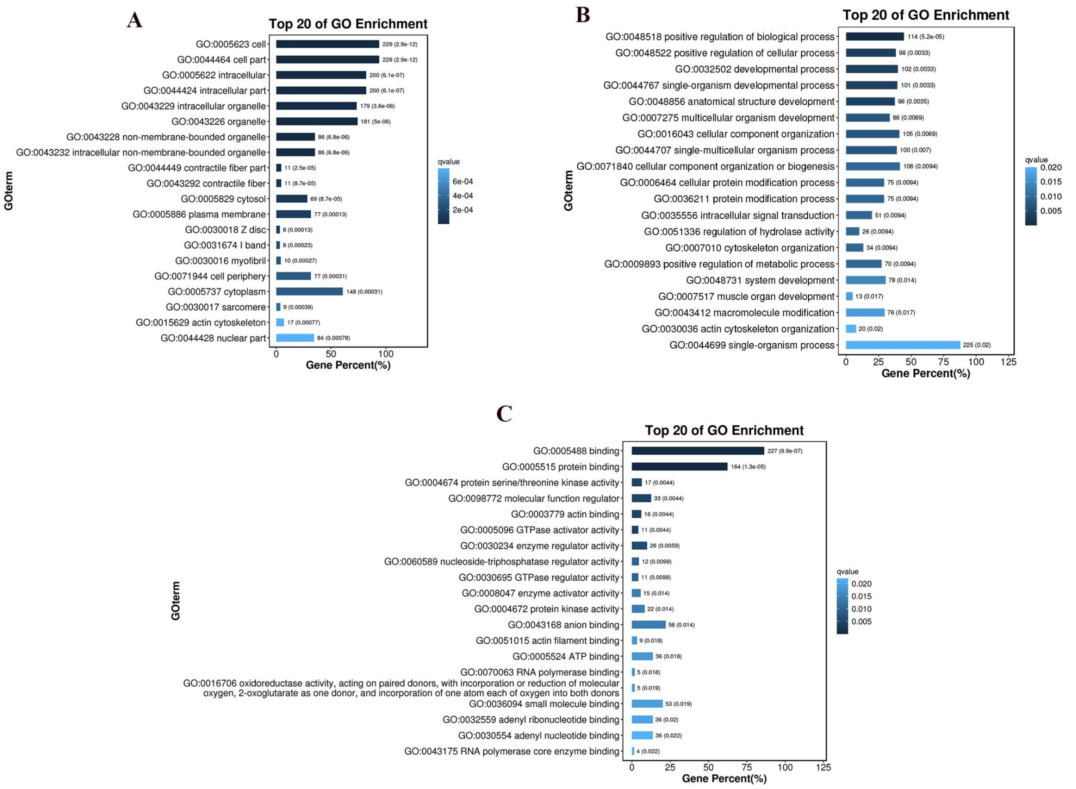

Supplementary Figure S2. GO enrichment analysis of DMEGs. A Top 20 of GO enrichments in Cellular Component. B Top 20 of GO enrichments in Biological Process. C Top 20 of GO enrichments in Molecular Function

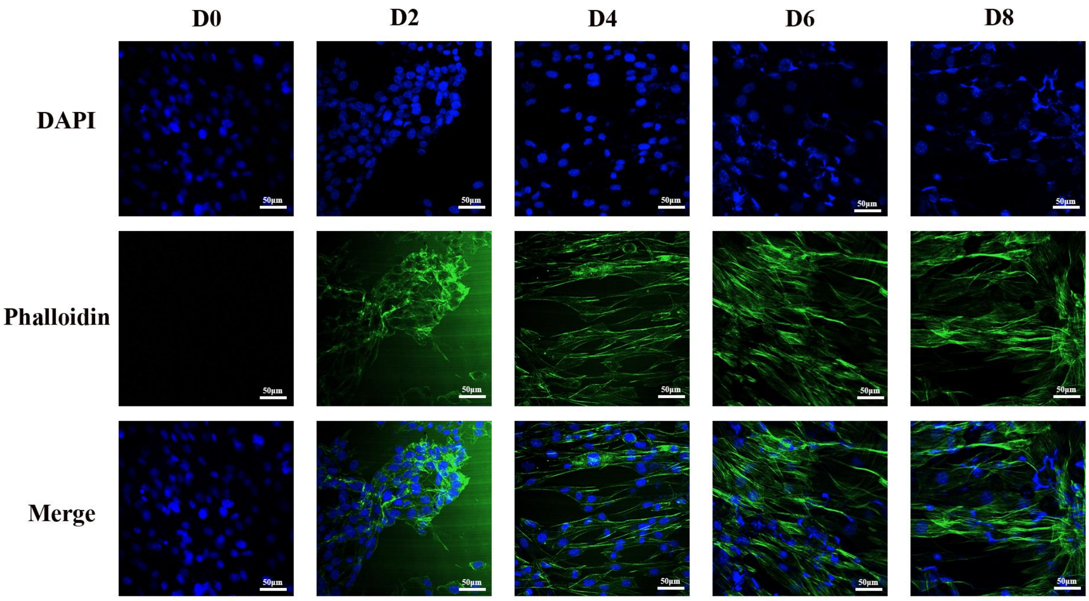

Supplementary Figure S3. The formation of the cytoskeleton. The magnification of the picture is 20X, that is, the magnification of the objective lens is 20 times

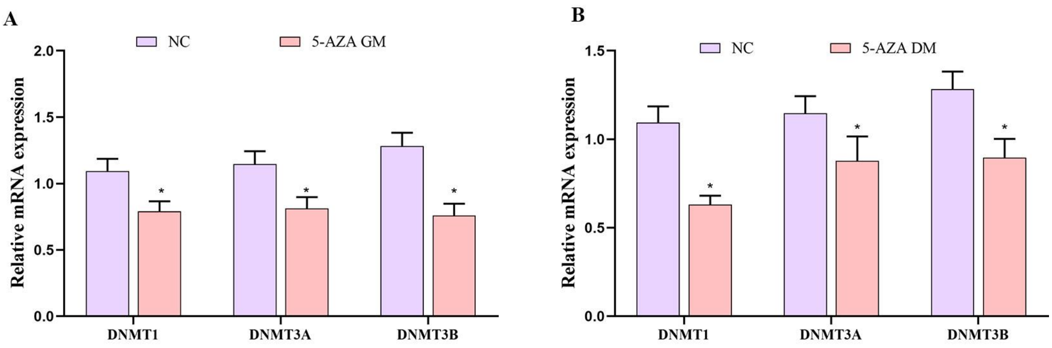

Supplementary Figure S4. Changes in the expression levels of methyltransferases after 5-AZA treatment of cells. A Expression levels of DNA methyltransferases during the GM phase. B Expression levels of DNA methyltransferases during the DM phase. The data are shown as the mean ± SD (n = 3), * $P < 0.05$.

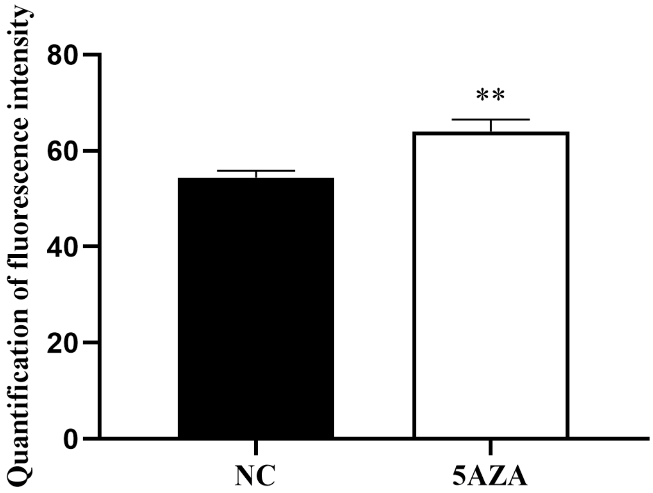

Supplementary Figure S5. Quantification of Figure 7B. The data are shown as the mean ± SD (n = 3), **, $P < 0.01$.

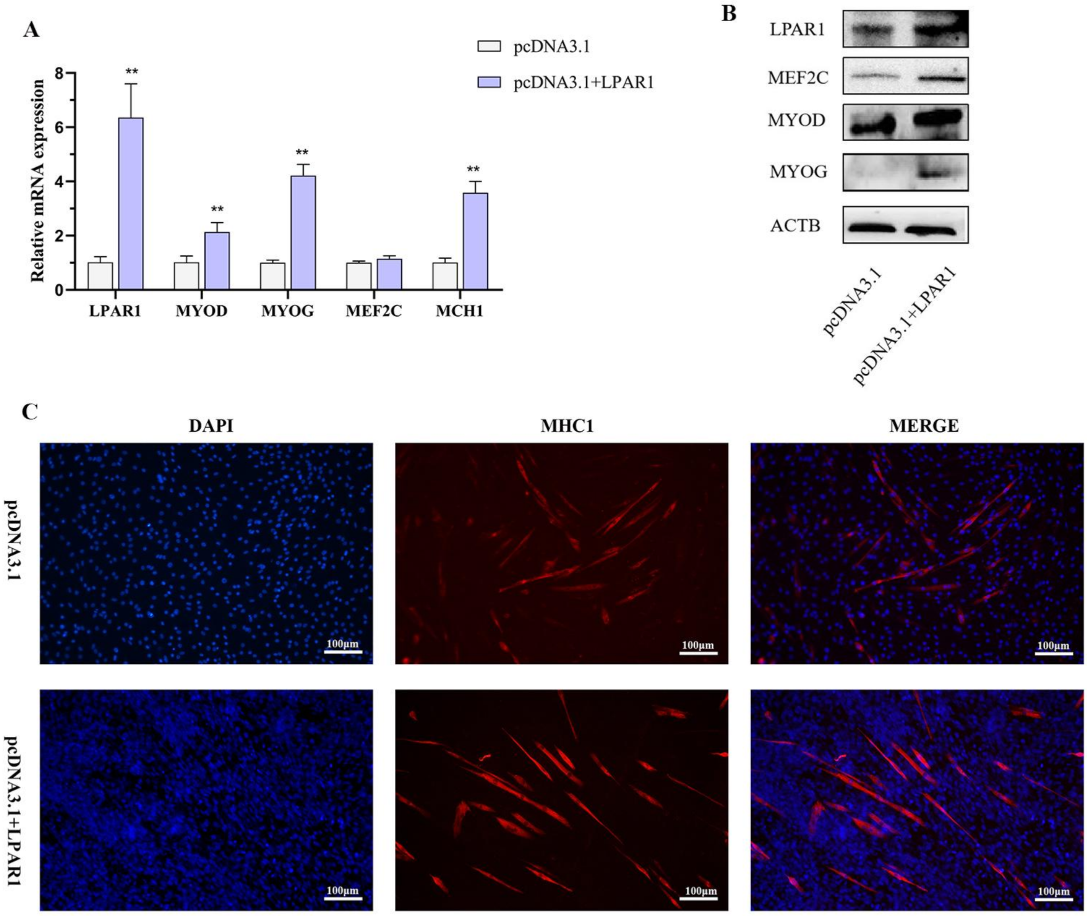

Supplementary Figure S6. Overexpression of *LPAR1* promotes differentiation of C2C12 myoblasts. A Expression of myogenic marker genes in C2C12 cells after overexpression of *LPAR1*. B Protein levels of myogenic marker genes in C2C12 cells after overexpression of LPAR1. C Immunofluorescence detection of myotube formation in C2C12 cells after overexpression of *LPAR1*. The magnification of the picture is 10X, that is, the magnification of the objective lens is 10 times. The data are shown as the mean ± SD ($n = 3$), **, $P < 0.01$

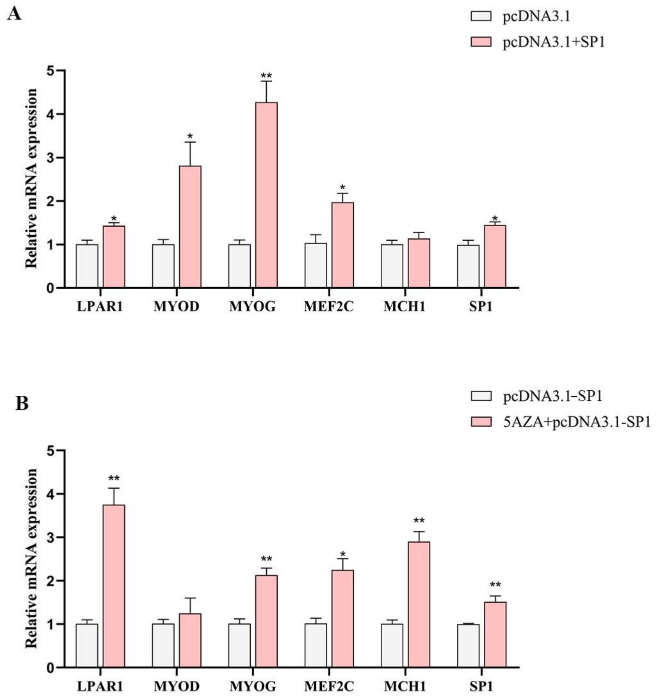

Supplementary Figure S7. Overexpression of SP1 and 5-AZA treatment promote myogenic differentiation in C2C12 cells. A Expression of myogenic marker genes in C2C12 cells after overexpression of SP1. B Expression of myogenic marker genes in C2C12 cells after 5-AZA treatment and overexpression of SP1. The data are shown as the mean ± SD (n = 3) *, P < 0.05, **, P < 0.01.
